# The role of environmental gradients and microclimates in structuring communities and functional groups of lizards in a rainforest-savanna transition area

**DOI:** 10.7717/peerj.16986

**Published:** 2024-04-26

**Authors:** Alan F. Souza-Oliveira, Gabriela Zuquim, Lidia F. Martins, Lucas N. Bandeira, Luisa Maria Diele-Viegas, Victor H.G.L. Cavalcante, Fabricio Baccaro, Guarino Rinaldi Colli, Hanna Tuomisto, Fernanda P. Werneck

**Affiliations:** 1 Coordenação de Biodiversidade, Instituto Nacional de Pesquisa da Amazonia, Manaus, Amazonas, Brazil; 2 Section for Ecoinformatics and Biodiversity, Department of Biology, Aarhus University, Aarhus, Denmark; 3 Department of Biology, University of Turku, Turku, Finland; 4 Department of Biology, University of Maryland, College Park, United States of America; 5 Departamento de Zoologia, Universidade de Brasília, Brasília, Distrito Federal, Brazil; 6 Departamento de Biologia, Universidade Federal do Amazonas, Manaus, Amazonas, Brazil

**Keywords:** Landsat, Thermal ecology, Climate change, Amazonia, Microclimate, Conservation

## Abstract

Environmental heterogeneity poses a significant influence on the functional characteristics of species and communities at local scales. Environmental transition zones, such as at the savanna-forest borders, can act as regions of ecological tension when subjected to sharp variations in the microclimate. For ectothermic organisms, such as lizards, environmental temperatures directly influence physiological capabilities, and some species use different thermoregulation strategies that produce varied responses to local climatic conditions, which in turn affect species occurrence and community dynamics. In the context of global warming, these various strategies confer different types of vulnerability as well as risks of extinction. To assess the vulnerability of a species and understand the relationships between environmental variations, thermal tolerance of a species and community structure, lizard communities in forest-savanna transition areas of two national parks in the southwestern Amazon were sampled and their thermal functional traits were characterized. Then, we investigated how community structure and functional thermal variation were shaped by two environmental predictors (*i.e.*, microclimates estimated locally and vegetation structure estimated from remote sensing). It was found that the community structure was more strongly predicted by the canopy surface reflectance values obtained *via* remote sensing than by microclimate variables. Environmental temperatures were not the most important factor affecting the occurrence of species, and the variations in ecothermal traits demonstrated a pattern within the taxonomic hierarchy at the family level. This pattern may indicate a tendency for evolutionary history to indirectly influence these functional features. Considering the estimates of the thermal tolerance range and warming tolerance, thermoconformer lizards are likely to be more vulnerable and at greater risk of extinction due to global warming than thermoregulators. The latter, more associated with open environments, seem to take advantage of their lower vulnerability and occur in both habitat types across the transition, potentially out-competing and further increasing the risk of extinction and vulnerability of forest-adapted thermoconformer lizards in these transitional areas.

## Introduction

Investigating the factors that determine the structure of biological communities and their composition has been a longstanding interest in ecology (Ricklefs, 1987; Huston, 1999), with many implications for understanding biodiversity patterns and conservation. Different processes acting at different spatial and temporal scales often interact to structure diversity and community assembly. Historical processes (*e.g.*, extinction, speciation, and dispersal in response to tectonic, eustatic, climatic, and oceanographic events) have a more substantial influence at the global and regional levels, while ecological processes (*e.g.*, species interactions, disturbance, and dispersal and drift along environmental gradients) have greater importance at local levels (Cavender-Bares et al., 2009; Algar, Kerr & Currie, 2011; Rapacciuolo & Blois, 2019). Local effects tend to be particularly relevant particularly for taxonomic groups with relative dispersal limitations and an intrinsic association with environmental variables. This is especially the case of groups such as lizards and other ectothermic organisms (Pianka & Vitt, 2003; Lewthwaite, Debinski & Kerr, 2017). At finer scales, factors such as edaphic characteristics (Whitney & Alonso, 1998; Pepper & Keogh, 2021), vegetation structure (Jellineck, Driscoll & Kirkpatrick, 2004; Tews et al., 2004) and microhabitat availability (Garda et al., 2013) are very influential because environmental heterogeneity can provide shelter, breeding and thermoregulation sites, and modulate biotic interactions. Moreover, environmental variations along ecological gradients or habitat transitions (*e.g.*, savanna-forest ecotones) bring about different selection pressures for maintaining populations (Chevin, Lande & Mace, 2010; Seebacher, White & Franklin, 2015), which generate regions of physiological and ecological tension that can interfere with the functional characteristics of the species and filter species occurrences (Liautaud, Barbier & Loreau, 2020).

Functional aspects of species have recently gained importance in community ecology studies (Mouillot et al., 2011), and are often combined with new tools and metrics to investigate species diversity and the encompassing environmental variations. Remote sensing, for instance, provides global and easily accessible data that serve as a proxy for vegetation structure and composition at different spatial scales (Tuomisto et al., 2003a; Tuomisto et al., 2003b; Tuomisto et al., 2019; Turner et al., 2003; Salovaara et al., 2005; Rocchini et al., 2018). On the other hand, microclimate variables that are remotely inferred using modeling (Algar, Morley & Boyd, 2018) or directly measured on the ground with data loggers (Alvarenga et al., 2017) can provide greater refinement and accuracy in quantifying microhabitat variations (Milling et al., 2018). Together, functional traits, remote sensing, and microclimate variables can help to better characterize species-environment interactions and distribution of organisms (Petchey & Gaston, 2002; Maximiano et al., 2020).

Temperature is a significant driver of biodiversity in multiple spatial and temporal dimensions (Waldock, Dornelas & Bates, 2018) since it directly affects species distribution and physiological traits, and the activity, growth, and reproduction of individuals (Savage et al., 2004; Angilletta, 2009). Lizards, for example, can vary the efficiency of their essential physiological functions according to environmental temperatures (Angilletta, 2009). The influence of environmental temperatures on physiological traits can be described using the shape of thermal performance curves. Their limits represent the minimum and maximum body temperatures supported by the individuals (*i.e.,* the critical temperatures that limit their physiological functions), and the range between these limits represents their thermal tolerance; and the maximal point in of the curve is related to the optimal temperature at which the animal can achieve its maximum performance (Huey & Slatkin, 1976; Deutsch et al., 2008; Grigg & Buckley, 2013; Taylor et al., 2020). Ectotherms modulate their body temperature according to the environment in two primary ways. Active thermoregulators, such as heliothermic organisms, actively maintain their body temperatures (Tb) within a specific range that is close to the optimum of their physiological performance (Huey & Slatkin, 1976; Angilletta, 2009), while thermoconformers passively align their Tb with the environmental temperature and usually present a broader range of optimal physiological temperatures (Angilletta, 2009). Environmental characteristics can also restrict or expand the species’ thermal abilities. For example, the low availability of sunlight spots in forest environments may cause species of forest lizards to have more restricted thermoregulation skills than those associated with open areas and even the thermoconformers (Huey et al., 2009; Piantoni et al., 2019).

The effects of climate change might be negative (*e.g.*, lead to range loss, local extinction), positive (*e.g.*, cause range expansion), or neutral for lizard species worldwide (Diele-Viegas et al., 2020) and may end up enabling warm-adapted, open-habitat competitors to invade forests and change community dynamics (Huey et al., 2009). Due to climate change, the thermal limits of tropical lizards will probably be exceeded in the following decades, making them and the tropical region particularly vulnerable to climate change (Deutsch et al., 2008; Sinervo et al., 2010; Diele-Viegas et al., 2020). Additionally, assessing the warming tolerance of these species can serve as a crucial proxy for evaluating vulnerability at both the population and community levels (Anderson, Meiri & Chapple, 2022). Therefore, expanding the understanding of the diversity of lizards and how physiological challenges in forest-savanna ecotones affect community assembly can help forecast the impacts of climate change on tropical biodiversity. The Amazon basin is a large mosaic composed of diverse landscapes (Tuomisto et al., 2019; Silva-Souza & Souza, 2020) that range from flooded and non-flooded forests to open ecosystems such as white-sand ecosystems (Adeney et al., 2016; García-Villacorta, Dexter & Pennington, 2016) and savanna enclaves (Ab’Sáber, 2002). This heterogeneity of habitats favors a high diversity of species in various taxonomic groups, and promotes the emergence of subpopulations through habitat specificity (Tuomisto, Ruokolainen & Yli-Halla, 2003; Pomara, Ruokolainen & Young, 2014; Menger et al., 2017). Nevertheless, it is unknown how species composition and thermal traits of species at local scales are related to micro- and macro-climatic variables. It may be particularly relevant in these Amazonian landscapes with forest, and savanna vegetation abruptly replaced.

Therefore, using new tools such as functional quantification *via* ecophysiology traits and environmental classification through remote sensing and micro-climate variables, we seek to test the following hypothesis and predictions: (i) The high environmental variation hypothesis suggests that the local occurrence of species and the assembly of lizard communities along a natural forest-savanna gradient in the Amazon are influenced by the high environmental heterogeneity. In line with this hypothesis, we predict that both micro-climatic and canopy reflectance variables will explain the turnover of lizard species composition, and indicate the influence of ecological factors on species assembly; (ii) The thermal trait variation hypothesis proposes that there are variations in these functional features across communities along the forest-savanna gradient, and that this variation is related to the different environmental characteristics along this gradient. Based on this hypothesis, we predict that species that are restricted to savanna and forest areas will exhibit distinct thermal functional variations, specifically, forest lizards, due to their more limited thermoregulation skills; (iii) The final hypothesis is the variation in vulnerability hypothesis, which suggests that there are implications for climate change vulnerability at the community level, and that these implications differ between savanna and forest communities. Consistent with this hypothesis, we predict that community vulnerability, assessed by species’ warming tolerance (WT), varies across habitats. In particular, given the higher prevalence of thermoconformers in forest environments, we expect them to display greater vulnerability to climate change compared to thermoregulatory species, which are more commonly found in savanna areas.

## Methods

### Study area

**Figure 1:**
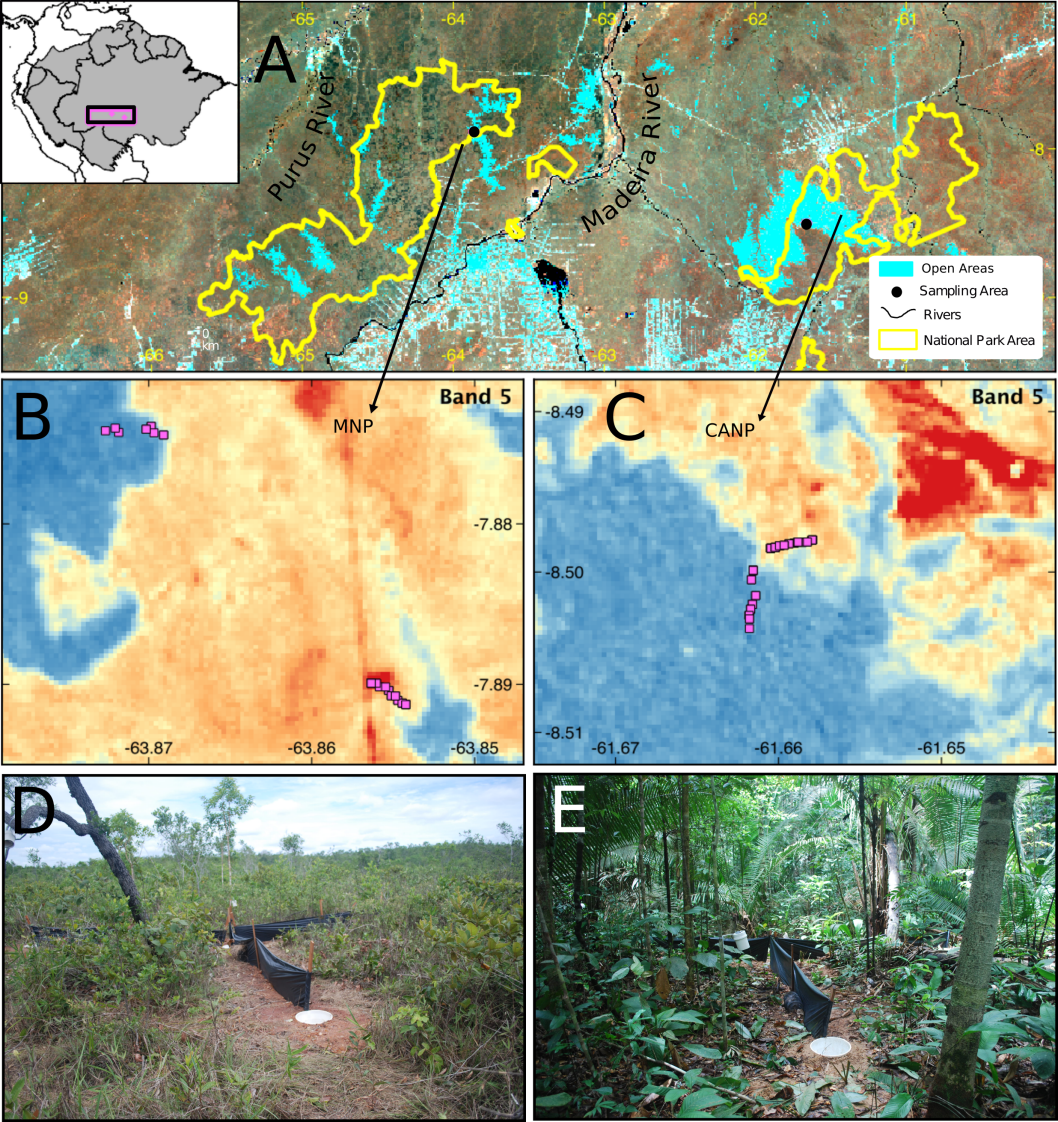
Sampling area in different perspectives and images of the trap set in the two sampled habitat types. (A) Location and limits of the Campos Amazônicos National Park (CANP) and the Mapinguari National Park (MNP). (B–C) Design and arrangement of plots in the different types of habitats in each of the parks based on the reflectance values of Landsat Band-5. The reddish tones represent open areas, and the blueish one indicates forest areas; the pink squares are the location of the pitfall traps. Pitfall traps in savanna (D) and forest (E) environments.

This study was carried out in two protected areas of the southwestern Brazilian Amazon: the Campos Amazônicos National Park (CANP) and the Mapinguari National Park (MNP) (Fig. 1). CANP is on the right (eastern) bank of the Madeira River in the municipality of Manicoré, Amazonas (8°30′S, 61°40′W) and has a total area of 961,318 ha (ICMBio, 2009). The area was sampled for 15 days in November 2017, at the beginning of the rainy season. The landscape consists mainly of extensive natural savannas, which consist of scattered trees amid a layer of grasses and shrubs. These savannas are presumably relics from the expansion of the Cerrado over the Amazon during the Quaternary interglacial periods (Josué Azevedo et al., 2020). The CANP also includes smaller areas of closed-canopy *terra-firme* forests, which are characterized by larger trees and a shaded understory. Deforestation due to livestock farming, gold mining, logging, and fire has impacted some areas of the park and its surroundings (ICMBio, 2009). With about 1,776,914 ha, the Mapinguari National Park (MNP), located on the left bank (western) bank of the Madeira River in the municipality of Humaitá (7°53′S, 63°51′W), was also sampled over 15 days in November and December 2017, at the beginning of the rainy season. MNP also features extensive savannas that, unlike those of CANP, are associated with geological events that affected sedimentation patterns, thus creating sharp edaphic boundaries (Rossetti et al., 2019). MNP faces similar threats as a result of current human pressure.

### Sampling

In each area, the lizard community was sampled using pitfalls traps. Each trap consisted of four 35 L buckets buried at ground level forming a “Y” shape, with one bucket at each end and another in the center, interconnected by 6 m-long and 0.8 m-high black tarp drift fences (*e.g.*, Werneck, Colli & Vitt, 2009; Garda et al., 2013). To maximize lizard captures, funnel traps (1 × 0.3 × 0.6 m minnow traps) were placed along the drift fences, one at each side, totaling six funnel traps. Hereafter, each set of pitfall and funnel traps is referred to a sampling plot (Fig. 1). In total, 25 plots were installed in each area along a transect, at least 50 m apart, to avoid spatial autocorrelation. To sample the environmental gradients in each park, approximately half of the plots were installed in open areas and the other half in *terra-firme* or lowland forest areas.

All individuals collected in the field were euthanized after collection of thermal traits (see below) using a lethal injection of thiopental. Following this, all specimens were fixed in 10% formalin and stored in 70% alcohol. Vouchers were deposited in the Amphibians and Reptiles Collection of the National Institute for Amazonian Research (INPA-H) and tissue samples were deposited in the Genetic Resources-*Herpetofauna* Collection (INPA-HT). All specimens were collected under permit # 57707-2, which was issued by ICMBio.

### Predictor variables

#### Micro-climatic variables

In each sample plot, a data-logger (Onset HOBO U23 Pro v2 temperature/relative humidity data logger) was installed to record the air temperature and relative humidity every minute during the 15-day sampling period. Data were collected as previously described in Alvarenga et al. (2017). In general, 14 micro-climatic variables were derived of which seven are related to humidity, such as absolute maximum relative humidity (Hmaxa), absolute minimum relative humidity (Hmina), absolute standard deviation of relative humidity (Hsda), maximum relative humidity (Hmax), mean relative humidity (Hmean), minimum relative humidity (Hmin), standard deviation of relative humidity (Hsd). In addition, more seven were derived from air temperature absolute maximum temperature (Tmaxa), absolute minimum temperature (Tmina), absolute standard deviation of temperature (Tsda), maximum temperature (Tmax), mean temperature (Tmean), minimum temperature (Tmin), standard deviation of temperature (Tsd).

#### Remote-sensing environmental variables

A remote-sensing dataset of canopy surface reflectance was used as a proxy for floristic and edaphic patterns (Tuomisto et al., 2019; Van doninck et al., 2020; Zuquim et al., 2021). We used a Landsat TM/ETM+ image composite that is already available (Van doninck & Tuomisto, 2018). It is based on cloud-free observations acquired over the ten-year period 2000–2009. Bands 4, 5, and 7 were used since they had been deemed informative in earlier studies (Van doninck & Tuomisto, 2018; Van doninck et al., 2020). The median reflectance values corresponding to a 15 by 15-pixel window centered on the coordinates of each plot (450-m window, given the original image resolution of 30 m) were extracted for each band. Non-forested pixels were masked using an unsupervised k-means clustering with a post-classification interpretation based on visual inspection of spectral signature and spatial distribution. Median reflectance values were obtained separately for forested and non-forested pixels within the window, and the number of pixels in each category (out of the total of 225) was registered.

### Species functional traits

For each sampled species, thermal biology was characterized using at least five captured specimens. The individual body mass was measured with a scale and then the snout-vent length (SVL) was measured with a digital caliper to represent the body size. To estimate the preferential temperature (*T*_*pref*_), the lizards were placed on a wooden thermal gradient (1 m long and 40 cm wide) for 1 h. One end of the gradient had a frozen gel pack, and the other had a heating lamp (∼100 W full spectrum). The lizards’ body temperatures were recorded every 3–5 min, with a one mm thermocouple attached with tape to their abdomen and connected to a data logger and the *T*_*pref*_ was estimated as the mean of all records. The second experiment measured the critical minimum (*CT*_*min*_) and maximum (*CT*_*max*_) temperatures of the lizards. For this, the lizards were placed first in a container to avoid direct contact with the material and then the container was placed in an icebox (*CT*_*min*_) or a thermal box with a heating lamp (∼100 W) (*CT*_*max*_) until the animal lost its locomotor response capacity, *i.e.,* when the animal was placed in the supine position and could not return to the upright position. The body temperature measured at this moment was regarded as the *CT*_*min*_ or *Ct*_*max*_ (*e.g.*, Taylor et al., 2020). Finally, we subtracted the values for *CT*_*min*_ from *CT*_*max*_ (*CT*_*max*_−*CT*_*min*_) to obtain the thermal tolerance range (TTR), which represents the individuals’ thermal amplitude, and also subtracted the mean *CT*_*max*_ from the mean maximum ambient temperature (*CT*_*max*_−*T*_*max*_) to obtain the warming tolerance (Deutsch et al., 2008). Ecophysiology data was collected following standard protocols (*e.g.*, Angilletta, 2009; Diele-Viegas et al., 2018; Pontes-da-Silva et al., 2018; Caetano et al., 2020), under authorizations from the Commission of Ethics and Animal Use of the University of Brasília (33786/2016) and INPA (029/2014). Species were classified as thermoregulators or thermoconformers based on Diele-Viegas et al. (2018). We did not classify any species, nor did we use any criteria based on our data to classify the thermoregulation mode of the species.

### Statistical analyses

To describe the variations in microclimate and canopy reflectance among the plots along the forest-savanna transition, a principal components analysis (PCA) of the correlation matrix was used.

The Bray–Curtis dissimilarity index was calculated based on abundance data in order to investigate dissimilarity in species composition within and between the two sampled areas. This measure represents the compositional turnover between the two sites and varies from 0 (the two sites have the same composition) to 1 (the two sites are entirely different and do not share any species). However, when calculated for two plots that do not share species, the index result will necessarily be equal to one, no matter how large the actual dissimilarity between them is. To mitigate this problem, the step-across or extended dissimilarity method was used (De’ath, 1999). Measures of extended dissimilarity can be obtained through one or more intermediate sampling units that share species with the non-species sharing samples. The value is calculated by adding the dissimilarity of these intermediate sample units. This procedure allows the dissimilarity between samples that do not share species to continue to grow as the ecological distance between them increases and facilitates the interpretation of ecological gradients in high-turnover data (De’ath, 1999; Tuomisto, Ruokolainen & Ruokolainen, 2012).

To investigate the first hypothesis of high environmental variation, a principal coordinates analysis (PCoA) was performed to visualize the composition dissimilarities as quantified using the extended Bray–Curtis dissimilarity, both for the two areas/national parks together and for each area separately. Multiple regressions were also performed to investigate which group of environmental variables (*i.e.,* micro-climatic or canopy reflectance) best explain the compositional turnover of the lizards (the first two axes of the PCoA), using the stepwise method based on the significance of the variables. To avoid redundant variables in the models, the correlation among all variables within each class of predictors (micro-climatic and vegetation structure estimated from canopy reflectance) was calculated. Pairs of variables with Pearson’s R of >0.7 were evaluated and subsequently removed or maintained in the model considering previous knowledge of lizard ecology and species-environmental relationships. After assessing the Pearson correlation among environmental predictors, four variables were retained for the subsequent multiple regressions: two micro-climatic (*T*_*min*_ and *H*_*max*_) variables and two variables based on canopy reflectance (Landsat Band 4 and Band 5) variables.

In order to investigate the second hypothesis regarding the variations in thermal traits, a PCoA based on Euclidean distance was used to summarize changes in the functional dissimilarity of the thermal traits for the lizards along the forest-savanna gradient. To this end, the community weighted mean (CWM) of each functional trait *T*_*pref*_, *CT*_*min*_, and *CT*_*max*_ was first calculated as the mean of the functional traits of the species weighted by the frequency of the species in each plot, thus enabling an evaluation of functional dissimilarity comparable to taxonomic dissimilarity. In addition, a PERMANOVA was used using the first two axes of the PCoA analyzed with CWM data as a function for both habitat types (forest-savanna) and the two national parks (MNP and CANP). These analyses were performed using the *vegan (Oksanen et al., 2019)* and *FD* packages (Laliberté, Legendre & Shipley, 2015) of the R statistical environment (R Core Team, 2020).

To investigate the third and last hypothesis regarding the variation in vulnerability and analyze the pattern of warming tolerance (WT) and the range of thermal tolerance (TTR) of the species, a Shapiro test was performed to analyze the normality of predictive parameters (*i.e.,* means of TTR and WT). The Shapiro test was also performed to assess whether they differ between habitat type and thermoregulation mode. When normality could not be assumed, a non-parametric Kruskal–Wallis test of differences between habitats and thermoregulation mode was performed. Finally, these results were used to qualitatively assess the vulnerability of species to global warming in the sampled areas. These analyses were performed using the R statistical environment’s *base* package (R Core Team, 2020).

## Results

### Environmental variations: microclimates and vegetation structure

**Figure 2:**
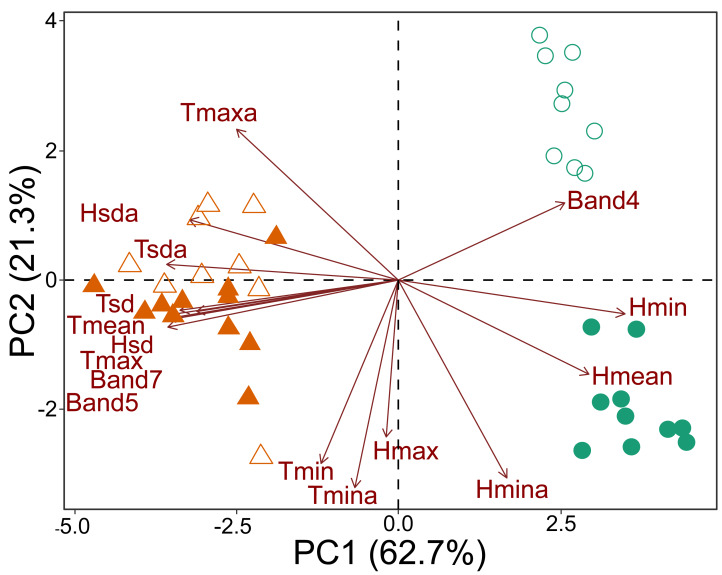
Multivariate ordination of the environmental variables, including micro-climatic and canopy reflectance/vegetation structure variables. Triangles: savanna plots; circles: forest plots. Open symbols represent sampling plots of the CANP and closed symbols represent plots of the MNP.

The principal component analysis based on microclimate variables and Landsat bands demonstrated that the plots could be divided into three groups. One group contained all the savanna plots, regardless of which national park was considered. The second group contained the forest plots of CANP, and the third group contained the forest plots of MNP (Fig. 2). All the forest plots were associated with high values of Landsat Band 4 and mean (*H*_*mean*_) and minimum (*H*_*min*_) relative humidity. When considering each national park that was sampled, a dichotomy associated with PC axis 2 axis was apparent (Fig. 2). The values of minimum (*T*_*min*_) and absolute minimum (*T*_*mina*_) temperature, and maximum (*H*_*max*_) and minimum absolute (*H*_*mina*_) humidity presented the strongest contributions. The forest plots of CANP had higher Band 4 reflectance values and lower minimum temperatures than the forest plots of MNP. In turn, savanna plots were more associated with high maximum (*T*_*max*_) and mean (*T*_*mean*_) temperatures, as well as with high reflectance values of Landsat Bands 5 and 7. The savanna plots were characterized by higher values of absolute maximum temperature and higher temperatures.

### Taxonomic composition and community structure

A total of 149 individuals were captured in both national parks, representing 17 lizard species in eight different families. *Kentropyx altamazonica* (35 individuals), followed by *Cercosaura olivacea* (25) and *Copeoglossum nigropunctatum* (24), were the most common species (Table 1). Despite two-thirds of the individuals originating from savanna plots (savanna: 100 individuals, forest: 49), the forest plots exhibited a higher species count (savanna: 10, forest: 12). However, it is crucial to acknowledge that this result may be biased due to the difference in the number of plots between the savanna and forest habitats. CANP had a greater abundance and richness of species, with eight species occurring only in this area, of which four were exclusive of to the savanna plots (*Cercosaura eigenmanni*, *Hoplocercus spinosus*, *Manciola guaporicola* and *Salvator merianae*), three were exclusive to forest plots (*Anolis tandai, Iphisa elegans* and *Loxopholis percarinatum*), and one occurred in plots of both habitats (*Cercosaura anordosquama*) (Table 1). Two species were sampled only in the MNP, one in the savanna plots (*Ophiodes* sp.) and the other in the forest plots (*Chatogekko amazonicus*).

**Table 1:** Checklist of the species collected in the two parks (CANP, Campos Amazônicos National Park; MNP, Mapinguari National Park) with respective sampling abundances by habitat, thermoregulation mode, and the species’ habitats of occurrence. (TR) thermoregulator; (TC) thermoconformer. Thermoregulatory information follows Diele-Viegas et al. (2018).

**Taxon**	**PNCA**		**PNM**		**Thermoregulation mode**	**Habitat of occurrence**
	**Savanna**	**Forest**	**Savanna**	**Forest**		
**Squamata**						
**“Sauria”**						
**Dactyloidae**						
*Anolis fuscoauratus* (D’Orbigny, 1837)	–	1	–	3	TC	Forest
*Anolis tandai* (Ávila-Pires, 1995)	–	2	–	–	TC	Forest
**Diploglossidae**						
*Ophiodes* sp.	–	–	2	–	TC	Open areas
**Gymnophthalmidae**						
*Cercosaura anordosquama* (Sturaro et al. 2018)	1	3	–	–	TC	Forest
*Cercosaura olivacea* (Gray, 1845)	16	1	8	–	TC	Open areas
*Cercosaura eigenmanni* (Griffin, 1917)	1	–	–	–	TC	Forest
*Iphisa elegans* (Gray, 1851)	–	1	–	–	TC	Forest
*Loxopholis percarinatum* (Müller, 1923)	–	2	–	–	TC	Forest
**Hoplocercidae**						
*Hoplocercus spinosus* (Fitzinger, 1843)	3	–	–	–	–	Open areas
**Scincidae**						
*Copeoglossum nigropunctatum* (Spix, 1825)	–	2	19	3	TR	Forest/Open areas
*Manciola guaporicola* (Dunn, 1935)	4	–	–	–	–	Open areas
**Sphaerodactylidae**						
*Chatogekko amazonicus* (Andersson, 1918)	–	–	–	4	TC	Forest
**Teiidae**						
*Ameiva ameiva* (Linnaeus, 1758)	2	–	16	4	TR	Forest/Open areas
*Kentropyx altamazonica* (Cope, 1875)	25	8	1	1	TR	Open areas
*Kentropyx calcarata* (Spix, 1825)	–	9	–	2	TR	Forest
*Salvator merianae* (Duméril & Bibron, 1839)	2	–	–	–	TR	Open areas
**Tropiduridae**						
*Plica umbra* (Linnaeus, 1758)	–	2	–	1	TC	Forest
**Total richness**	8	10	5	7		
**Total abundance**	54	31	46	18		

**Figure 3:**
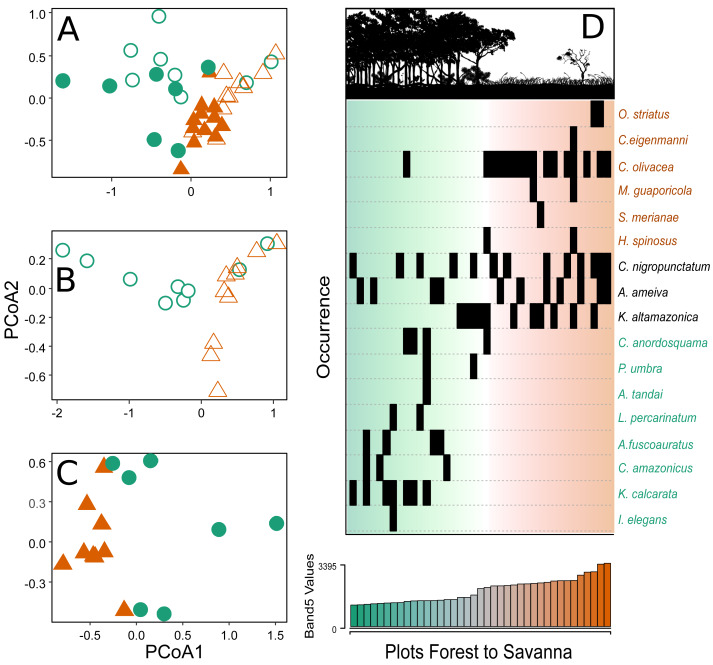
Principal coordinate analysis depicting the species turnover between plots along the environmental gradient. (A) Plots in the CANP and MNP, (B) plots in the CANP, and (C) plots fin the MNP. (D) Direct ordination of the species by occurrence along the gradient of Landsat Band 5 values. Triangles = savanna plots, circles = forest plots. Open symbols represent sampling plots in the CANP, and closed symbols represent plots in the MNP.

The PCoA analyses showed two distinct groups in terms of species composition when considering the environmental gradient, both when evaluating all the sampling plots of the two parks together (Fig. 3A) and plots from each park separately (Figs. 3B–3C). The savanna plots were more similar to each other in terms of species composition than the plots of the forest area.

After a single round of the multivariate stepwise multiple regressions, the simple model containing the variable Band 5 was selected (*P* < 0.001, *R^2^* = 0.335). This result indicates that canopy reflectance of short-wave infrared radiation (Band 5) is the best predictor of species turnover along the forest-savanna gradient when considering all park plots together (Fig. 3A). The direct ordination of species composition based on occurrence in the plots also demonstrated substantial turnover associated with values of the Landsat Band 5 (Fig. 3D).

### Thermal ecology

**Figure 4:**
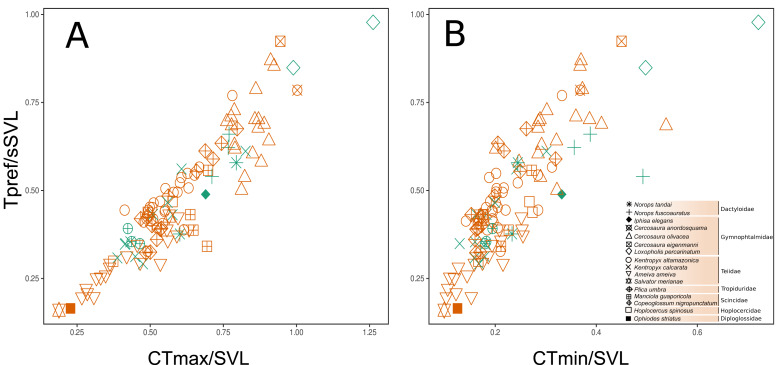
Functional space of the sampled communities based on the thermal traits of the species and family level. The preferred temperature (T_pref_) axis weighted by body size (SVL) varies correlated to the maximum critical temperature (*CT*_*max*_ and minimum critical temperature (*CT*_*min*_) axes, both weighted by body size (SVL). The weighting of the variables by body size (SVL) was used to remove the effect of this parameter on the thermal temperature traits. (A–B) is the space considering the savanna-forest transition (reddish = savanna; greenish = forest). The thermal trait values can be found in the Supplementary File.

A strong correlation was found between the three functional traits evaluated, in which *T*_*pref*_ and *CT*_*max*_ had the highest values (*r* = 0.921, *P* < 0.0001), followed by *CT*_*max*_ *vs. CT*_*min*_ (*r* = 0.825, *P* < 0.0001) and, finally, *T*_*pref*_ *vs. CT*_*min*_ (*r* = 0.817, *P* < 0.0001). This indicates that any possible selection of one thermal trait will likely affect the evolution of all those considered here. In addition, this collinearity could represent a shared phylogenetic history among the species. Despite the large overlap, there was a subtle difference between the two habitats in the functional strategies of individuals (Figs. 4A–4B). Except for *Loxopholis percarinatum,* species that occur in the forest plots have a range of lower to intermediate values for these key thermal physiology traits. On the other hand, species that occur in the savanna plots occupy a much wider trait space (Figs. 4A–4B). The species with lower values for *CT*_*min*_, *CT*_*max*_, and *T*_*pref*_ were *Ophiodes* sp. (Diploglossidae), *Salvator merianae, Ameiva ameiva, Kentropyx calcarata* (Teiidae), followed by species with intermediate values *K. altamazonica* (Teiidae), *Hoplocercus spinosus* (Hoplocercidade), *Copeoglossum nigropunctatum*, *Manciola guaporicola* (Scincidae), *Plica umbra* (Tropiduridae), *Anolis fuscoauratus, A. tandai* (Dactyloidae) and *Iphisa elegans* (Gymnophthalmidae). Finally, the species with the highest values for the three traits were *Cercosaura olivacea*, *C. anordosquama*, *C. eigenmanni* and *Loxopholis percarinatum* (Gymnophthalmidae) (Figs. 4A–4B). All ecothermal data, both weighted by SVL, mass, and the raw data for species and individuals are available in the supplementary material.

**Figure 5:**
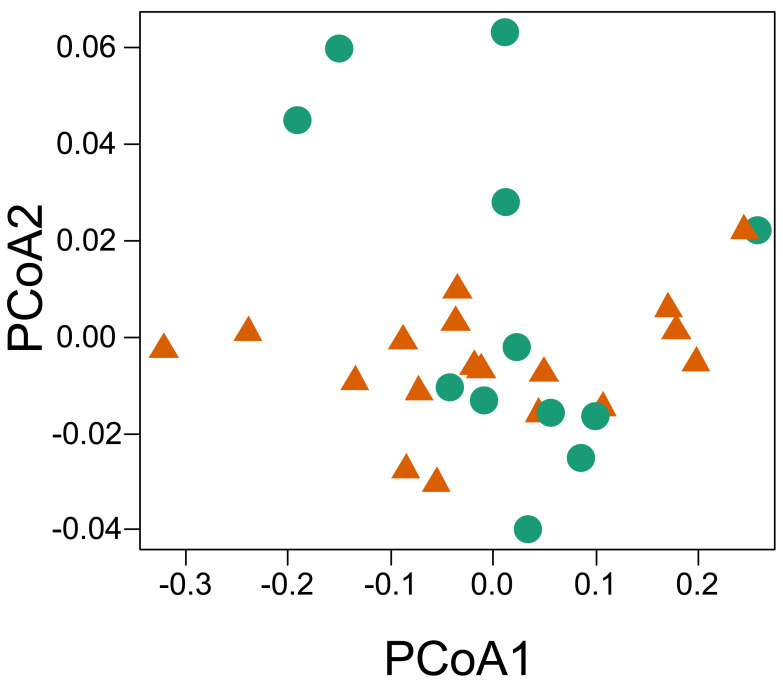
Principal coordinate analysis based on the dissimilarity of the community weighted means (CWM) of thermal traits of lizards from two parks in the southwestern Amazon. Red triangles represent savanna plots; green circles represent forest plots.

The principal coordinate analysis showed no clear pattern of dissimilarity between CWM values taking into account the two habitat types (Fig. 5). However, when we considered the same analysis and explored the dissimilarity of the functional traits between the two parks, we were able to recover a dissimilar pattern between them. When running the PERMANOVA, we were able to observe a significant relationship of dissimilarity between the two national parks with respect to the composition of functional traits of the sampled individuals (*P* = 0.013, *R*^2^ = 0.159, permutations 999).

**Figure 6:**
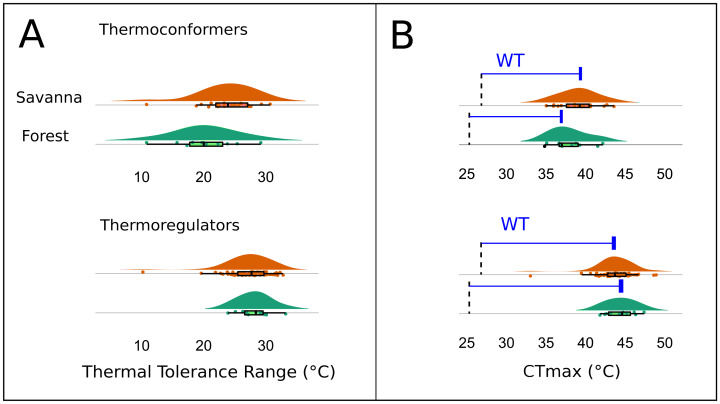
Thermal and warming tolerance of the lizard community across two habitat types and their thermoregulatory modes. (A) Thermal tolerance range (TTR) of the lizard species considering habitat type and thermoregulation mode. (B) Frequency of the critical maximum temperature (*CT*_*max*_) of the species considering the two types of habitats and the thermoregulation behavior. The dotted line indicates the mean maximum temperature recorded in the respective environments, and the difference between the bar and the curves is the warming tolerance (WT) (blue bar).

The Kruskal–Wallis test showed that there was no significant difference in mean TTR between the two types of habitats or between thermoregulation modes (Chi-squared = 20.953; *P* = 0.399; *df* = 20 and Chi-squared = 29.086; *P* = 0.3072; *df* = 26, respectively). However, the two thermoregulatory behaviors differed in the breadth of the thermal ranges (Fig. 6A). Thermoconformers (TC) had a much broader and more generalized TTR, regardless of habitat type; while, thermoregulators (TR) had a narrower TTR, varying slightly in more specific temperature values (Fig. 6A).

The *CT*_*max*_, when subtracted from the maximum temperature of the environment, resulted in a smaller margin for the thermoconformers than the thermoregulators (Fig. 6B). However, forest thermoconformers had a smaller safety margin for *CT*_*max*_ in relation to thermoregulators, indicating a narrow warming tolerance (Table 2). For thermoregulators, the type of habitat made no difference to the individual’s warming tolerance.

**Table 2:** Mean *CT*_*max*_ and maximum environmental temperature (*T*_*max*_) values and warming tolerance (WT) by habitat type and thermoregulation mode for lizard species in the two sampled parks.

	Thermoconformers		Thermoregulators	
	**Forest**	**Savanna**	**Forest**	**Savanna**
**MCTmax (°C)**	37.9	39.1	44.4	43.8
**Mtmax (°C)**	29.55	29.54	29.55	29.54
**WT (°C)**	11.35	9.56	17.85	14.26

## Discussion

### Environmental variations

The environmental transition along the savanna-forest ecotone corresponds to clear micro-climate and canopy surface reflectance differences in the sampled areas. Moreover, as shown by the variation along the PC 2 axis (Fig. 2), there is a clear distinction between the parks’ habitat characteristics. This result may be inherent due to differences in their geological formation histories (see Rossetti et al., 2019; Ruokolainen et al., 2019; Josué Azevedo et al., 2020), which would be responsible for a floristic and micro-climate turnover between both environments. In general, forest areas are cooler, with significant variation in air humidity and higher reflectance of the near-infrared Landsat Band 4. The low and highly variable relative humidity in these forest environments compared to the same habitat type in the center of the biome seems to be a characteristic of this habitat type in the southwestern portion of the Amazon, especially for areas that border savanna environments and are susceptible to fire (Brando et al., 2014). The savanna plots, in turn, are environments with higher temperatures, greater incidence of sunlight, greater stability in relative humidity, and higher reflectance of Landsat Bands 5 and 7 (Fig. 2).

In vegetated areas, the surface reflectance registered by satellites depends on the kinds of functional plant properties that typically vary among edaphically determined vegetation types, such as the degree of woodiness and the amount and structure of photosynthetic leaf tissue (Tuomisto et al., 2003a; Tuomisto et al., 2019; Serbin & Philip, 2020). Band 4, for example, registers wavelengths in the near infrared (0.77–0.90 µm), whose reflectance depends on photosynthetic activity and biomass, with the highest reflectance values associated with forest environments, in which trees accumulate more biomass than the smaller plants in open areas. Bands 5 (short-wave infrared, 1.55–1.75 µm) and 7 (also short-wave infrared, 2.09–2.35 µm) indicate leaf water content, which is typically lower in woody plants (such as trees) than in herbaceous plants (such as savanna grasses and sedges). (*e.g.*, Schwieder et al., 2016). Therefore, our results reinforce the use of these variables as good alternatives that more accurately provide the limit between the vegetative types of savanna and forest, in addition to demonstrating the variability that exists within these environments (Fig. 2).

### Composition of lizard species

The taxonomic composition showed an evident dissimilarity between forest and savanna habitats under both configurations of analysis (the assemblages of the two parks together and separately). It is important to consider that the low abundance in the forest habitats, with only 49 individuals, could cause bias in the results, mainly in regards to comparisons within the two habitats in the same park. Such structuring was represented by a strong pattern of species turnover along the canopy reflectance gradient, which was the variable that explained this pattern best (Fig. 3D). This result corroborates our expectation that the lizard community would show considerable species turnover along the forest-savanna transition. A similar result was reported by Vitt & Zani (1998) in a forest-savanna in the northern Amazon, and indicates that this could be a general pattern.

The influence of environmental variables, such as canopy openness, number of trees, density vegetation, elevation and soil structure in structuring ectothermic vertebrate communities on a local scale has been reported for the mid-upper Madeira River (*e.g.*, Garda et al., 2013; Dias-Terceiro et al., 2015; Peixoto et al., 2020). The fact that temperature is not the main variable or determining factor for lizard assembly is intriguing (Fig. 3), as temperature often affects several physiological and behavioral activities of lizards and ectotherms in general (Angilletta, 2009). However, the discussion of the role of temperature in the presence of ectotherms and lizards themselves is still a question to be further investigated. Kearney (2013), for example, comments that the activity time alone is not capable of determining the presence of an ectothermic species, while Caetano et al. (2020), in a macro-climatic approach, argues that such a parameter is a better predictor than the temperature variable alone. If we consider Band 5 an indicator of such functional aspects of the vegetation that may be important for the lizards and that, in addition, there is a large contribution of micro-climatic temperature variables, our result supports the idea that, despite not having a primary role, temperature has a greater contribution when the other factors that are correlated to it are taken into account. Vitt & de Carvalho (1995) also argue that climate alone is not determinant for the diversity of lizard species, especially for open areas, which have lower diversity that is inherently due to the lower structural diversity of the environment, and this may also explain the lower species richness that was found in the savanna.

Spatial heterogeneity of vegetation and vegetation cover are known to affect the occurrence and abundance of squamates in the tropics, as they are related to the availability of resources and sites for nesting, foraging, thermoregulation (Vitt & Zani, 1998; Vitt et al., 2007; Pringle, Webb & Shine, 2003; Masseli et al., 2019; Mesquita et al., 2015) and variation of light incidence, humidity and temperature (Pringle, Webb & Shine, 2003; Peixoto et al., 2020). The communities sampled herein are also affected by these factors since they are structured along a gradient of plant structure, which is represented by canopy reflectance, varying in the availability of open environments with a high incidence of light, temperature, and humidity, as well as forest environments with a lower incidence of direct light and milder temperatures. Furthermore, forest environments have a greater availability of microhabitats thanks to the vertical stratification; whereas, in the Cerrado biome (open areas), habitat stratification occurs horizontally over various types of vegetation (Colli, Bastos & Araujo, 2002; Gainsbury & Colli, 2003; Nogueira, Valdujo & França, 2005). The higher lizard richness that was recorded in forest plots (Table 1) may result from the fact that typical forest species do not usually disperse to savanna areas (Vitt & de Carvalho, 1995), while heliotherms from open areas, such as *Ameiva ameiva*, *Copeoglossum nigropunctatum*, and *Kentropyx altamazonica,* often use forest clearings formed by treefalls and forest edges, thus increasing the species richness of the forest habitat.

The difference in species richness and distribution between the parks may be related to historical factors that affect speciation and extinction, such as the different geological histories of these environments, especially in the savannas. Gainsbury & Colli (2003), for example, argue that the lizard assemblages from savanna enclaves in the southwestern Amazon are structured by stochastic factors, particularly extinctions and isolation of these areas caused by the retraction of the Cerrado and the expansion of the forest in the Holocene. They also demonstrate that enclaves that are geographically distant from the core of the Cerrado have fewer species than those closer to the ecotone. Our results mirror this pattern, since the species richness of savanna areas in the CANP is higher than what is found in the MNP, which is further away from the core of the Cerrado and is also separated by the Madeira River, a critical biogeographic barrier for many species (Peixoto et al., 2020).

### Thermal ecology and the implications of climate change for the community

No dissimilarity pattern of lizard thermal functional variation was found between the two forest-savanna habitat types. On the other hand, we did encounter a significant difference in dissimilarity between the national parks. Although it seems to contradict initial predictions, this result makes sense if one considers that *CT*_*max*_, *T*_*pref*_, and *CT*_*min*_ are parameters that are normally under phylogenetic conservatism (Grigg & Buckley, 2013), as well as being correlated with each other and sharing phylogenetic histories (Diele-Viegas et al., 2018; Fig. 5). The thermal traits do not seem to be influenced by the environmental filtering promoted by the specific local conditions of the two habitat types (savanna-forest). Another possibility would be that the environmental filters are similar in both habitats, which is common at the local scale (Kraft et al., 2015). However, on a regional scale, the significant difference in dissimilarity of the thermal traits used to compare the two parks possibly reflects the influence of the inherent environmental filters of the different climatic and geological events that shaped the environment of these two parks (see Josué Azevedo et al., 2020; Rossetti et al., 2019). We encountered an apparent pattern of structure in the species’ functional space at the family level, which reinforces a possible role of evolutionary history on the thermal traits of the lizards (Bodensteiner et al., 2020). This may be better observed with the inclusion of the taxonomic family as a structuring factor. It is important to report that we did not explicitly test historical factors, but we recommend such approaches in further work, given the trend found in our results. On a regional scale, the preferential temperature (*T*_*pref*_) has been reported to be more affected by factors other than phylogeny, such as environmental temperatures, which makes it more prone to show greater phenotypic plasticity along environmental transitions (see Diele-Viegas et al., 2018; Pontes-da-Silva et al., 2018). However, on a local scale, we did not observe variations between habitats in *T*_*pref*_ among individuals of the species in both forest and savanna plots (*i.e., C. nigropunctatum, A. ameiva, K. altamazonica*). This result may reflect the high correlation between thermal features analyzed or their more active thermoregulation behavior attenuating this variation as they move between the two habitat types (Huey et al., 2012).

Tropical lizard species are considered especially vulnerable to global warming, as they experience environmental temperatures that are already close to or exceed their optimum temperatures and maximum performance (Deutsch et al., 2008; Huey et al., 2009; Clusella-Trullas & Chown, 2014). Furthermore, species adapted to warmer tropical environments can be asymmetrically affected in comparison to those associated with slightly cooler tropical climates, a typical scenario of transitional environments, such as savanna-forest transitions (Huey et al., 2012). In this context, thermoconformers, which were more common in the forest with a mild thermal habitat, are more vulnerable to heat than thermoregulators, which are more common in savannas where the habitat is warmer (Huey et al., 2009). Nonetheless, there is some evidence regarding thermoregulator species buffering their vulnerability to heating, for instance, a macroscale study of the thermal traits in different populations of *K. calcarata* across the Amazon showed higher values for *CT*_*max*_ to for populations close to the Cerrado ecotone (open areas) and lower values in the same species located in the core of the biome (Pontes-da-Silva et al., 2018). We found the same patterns for the same species, but on a local scale in which the *CT*_*max*_ reached 44.3 °C close to the savanna habitats; while Pontes-da-Silva et al. (2018) recorded values below 44 °C in the same ecotone.

We did not encounter a significant difference for the TTR of individuals between forest and savanna habitats or between the two main thermoregulation modes, thus reflecting the weak environmental filtering for thermal traits discussed previously. However, in line with expectations (*e.g.*, Huey et al., 2012; Grigg & Buckley, 2013), thermoregulator lizards showed a narrower pattern of the thermal range when compared to the thermoconformers, which is a difference that implies different community responses to global warming. Active thermoregulation can mitigate climate change effects at the local scale, which vary according to the environments’ thermal heterogeneity, and is more limited in forest habitats than in open areas. Environments that are thermally more homogeneous, such as forests, represent fewer possibilities for variation in the lizards’ activity period (Fitzgerald, Shine & Lemckert, 2003; Kiefer, Van Sluys & Rocha, 2007). Moreover, the warming tolerance (WT) is also known to be affected by the environment’s thermal variability, whereby less heterogeneous environments have low WT. Consequently, thermoconformers should be more affected, as they are more frequent in the forest and have less tolerance capacity than open-habitat thermoregulators (Deutsch et al., 2008; Huey et al., 2012). Our results indicated this pattern, since we found a lower WT for thermoconformers, thus reiterating expectations of their greater vulnerability.

Although thermoconformers were the most common in the forest plots, there was no difference in lizard WT between the forest and savanna plots. This result indicates that assemblages in forest areas might be already suffering from the influence of assemblages from surrounding open habitats (Brando et al., 2014; Sales, Galetti & Pires, 2020). Huey et al. (2012) warned that in forest lizards heat stress would trigger another type of stress called biotic stress, whereby species from open areas will occupy niches to escape from warming and benefit from forest structure changes. Our observations of some species from open environments in forest plots, but not the reverse, apparently support Huey’s warning. Further studies are needed in order to assess the impact of global warming on community dynamics and the likelihood of “savannization” of tropical lizard communities at broader scales (Sales, Galetti & Pires, 2020).

Finally, we conclude that lizard communities in Amazonian forest-savanna transitions tend to be ecologically structured by habitat heterogeneity. The species’ thermal ecology traits are not dissimilar between the environmental filters of forest-savanna gradient and seem to be more associated with their evolutionary history. The same is not true when evaluating the thermoregulatory modes and their parameters of thermal and warming tolerance. However, all these findings proved to be connected when we consider the responses of lizard species to global warming. Firstly, the forest structure, which is very important for the taxonomic composition, may already be suffering from changes (Algar, Morley & Boyd, 2018; Sales, Galetti & Pires, 2020) and, thus, benefits species from open areas, which find more favorable conditions in these environments, such as a refuge from overheating. Secondly, the thermal tolerance range and the warming tolerance demonstrate an asymmetry between the thermoregulation modes and, consequently, in the vulnerability to climate change, which is greater for thermoconformers than thermoregulators. This pattern leads to biotic stress on forest habitats and their associated species which are mostly thermoconformers, and potentially increases their vulnerability and risk of extinction.

## Supplemental Information

10.7717/peerj.16986/supp-1 Supplemental Information 1 Occurrence of the species collected by traps All traps from one to ten (1-10) are relative to traps located in the savannah areas in both parks. (*e.g.*, PNM01, PNM2... PNM10; and PNCA01, PNCA02... PNCA10). The traps allocated in forest habitats are from 11 to 25 (11-25), also in both parks (*e.g.*, PNM11, PNM12... PNM25; and PNCA11, PNCA12... PNCA25).

10.7717/peerj.16986/supp-2 Supplemental Information 2 Average values of functional traits by traps

10.7717/peerj.16986/supp-3 Supplemental Information 3 Functional traits of all sampled individuals The functional traits that appear in the “prop” in the title, that is proportional to: mass or svl (length). When there is no “prop” in the parameter title it means that it is the raw data.

## Additional Information and Declarations

### Field Study Permissions

The following information was supplied relating to field study approvals (i.e., approving body and any reference numbers): Field experiments were approved by the Instituto Chico Mendes de Conservação da Biodiversidade - ICMBio (#57707).

### Data Availability

The following information was supplied regarding data availability: The raw data are available in the Supplemental Files and at OSF: de Souza Oliveira, Alan F. “The Role of Environmental Gradients and Microclimates in Structuring Lizard’s Community and Functional Groups at a Rainforest-savanna Transition.” OSF, 3 Jan. 2023. Web. https://doi.org/10.17605/OSF.IO/2CERX.
